# Reliability of Quadriceps and Hamstring Soft Tissue Measures Using Dual-Energy X-Ray Absorptiometry (DXA) Scans

**DOI:** 10.3390/jimaging11050127

**Published:** 2025-04-22

**Authors:** Trey R. Naylor, Mariana V. Jacobs, Cameron J. Elder, Michael A. Samaan, Jody L. Clasey

**Affiliations:** 1 Department of Kinesiology and Health Promotion, University of Kentucky, 216 Seaton Center, Lexington, KY 40502, USA; trey.naylor@uky.edu (T.R.N.); mariana.jacobs@uky.edu (M.V.J.); cameron.elder@uoflheath.org (C.J.E.); michael.samaan@uky.edu (M.A.S.); 2 Department of Pediatrics, University of Kentucky, Lexington, KY 40502, USA

**Keywords:** body composition, dual-energy X-ray absorptiometry, mineral-free lean, region of interest

## Abstract

**Purpose:** The purpose of this study was to determine the intra- and interrater reliability of quadriceps and hamstring soft tissue measures using DXA scans. **Methods:** A total of 44 subjects (23 males) participated in this study. The first total body DXA scan was performed in the standard anterior/posterior scanning position, followed by two additional total body scans while the subjects were lying on their left and right sides with the leg of interest extended and the contralateral leg bent. Unique regions of interest were created for analyses of mineral-free lean masses (MFL) using custom analysis software with manual tracing (by two investigators) of the quadriceps (QUADS) and hamstrings (HAMS) of the right and left thighs. Between–within repeated measure analysis of variance (ANOVA) was used to determine if there were significant differences among the MFL measures, while intraclass correlation coefficients (ICC) and coefficients of variation (CV) were used to assess the intra- and interrater reliability. **Results:** Between-group analyses revealed that Investigator 2 had small yet significantly higher mean differences for right QUADS (2346.6 ± 602.4 g vs. 2327.4 ± 587.9 g), left QUADS (2337.3 ± 581.9 g vs. 2312.8 ± 581.2 g), right HAMS (2655.9 ± 626.3 g vs. 2543.0 ± 593.5 g), and left HAMS (2686.1 ± 628.1 g vs. 2562.8 ± 596.5 g) when compared to Investigator 1. Intraclass correlation coefficients between (≥0.984) and within (≥0.992) raters were high for all MFL measures, with low variation across all MFL measures (≤1.62%). **Conclusions:** Despite having significant group mean differences, our results revealed strong and significant reliability, and we recommend that a single investigator analyze the scans twice and that the mean of the two measures be used for final reporting within a given study.

## 1. Introduction

Dual-energy X-ray absorptiometry (DXA) is a technology, originally introduced in 1987, that can produce total body and regional bone mineral content density (BMC; g) and bone mineral density content (BMD; g/cm^2^) measures [1,2]. Since its initial FDA approval in 1988, DXA has undergone many significant technological advances in software capabilities that have allowed the inclusion of soft tissue analyses, including absolute and relative fat and mineral-free lean (MFL) masses [3]. In addition, the inclusion of a custom analysis function in the existing software provides limitless possibilities for creating unique regions of interest (ROI) for both bone and soft tissue analyses.

Collectively, the newer DXA software (software version 14.10) functions provide researchers and clinicians with a method of determining total body and regional (both standardized and uniquely created) body composition measures. The DXA scan analysis has advantages over other body composition assessment methods because it requires minimal subject cooperation, the measurement time is rapid (a total body scan requires approximately 10 min to perform), the subject is exposed to minimal radiation (less than 1 mrem for a total body scan), and its less expensive and more widely available than computed tomography (CT) or magnetic resonance imaging (MRI) methods. Furthermore, DXA allows for both fat-free and MFL mass measures to be determined [2,4,5]

In clinical settings, DXA is used to assess the presence or risk of osteoporosis, to monitor the loss of lean mass due to aging or disease conditions and may provide visceral adipose tissue estimations [6,7,8]. Total body DXA scans completed in the standard anterior–posterior positioning provide measures of bone and soft tissue of the thigh using the custom analysis function. However, alternative positioning for total body scans is required if separate analyses of the quadriceps and hamstrings MFL masses are desired. Specifically, participants must have their total body scans performed while they are lying on their right or left sides with the measured leg extended and lying against the scan table. Since creating these unique ROI requires subjectivity while tracing the quadriceps and hamstring musculature, both intra- and interrater reliability measures should be determined [9,10].

These reliability measures may vary according to anatomical boundaries used for created ROI, software algorithms of the DXA scanner, and the manufacturer model [11]. Therefore, minimal variability within and between investigators is desirable to improve the accuracy when these measures are compared between participant populations when relationships between soft tissue and strength measures are desired, and when the effectiveness of intervention and rehabilitative strategies are assessed. Thus, this paper will determine the within (intra) rater and between (inter) rater reliability of quadriceps and hamstring soft tissue measures using a unique methodological analysis before providing concluding evidence that supports our findings. The proceeding of this paper is structured as follows: Section 2 presents the materials and methods, Section 3 outlines the results of the statistical analysis, Section 4 provides a detailed discussion of the findings and concludes the paper with final remarks and recommendations for future research.

## 2. Materials and Methods

**Subjects**. A convenience sample of 44 subjects (23 males) participating in ongoing clinical research studies requiring DXA scanning completed the requirements for this study. Exclusion criteria for this study included a positive pregnancy test at the time of DXA scanning or previous placement of lower limb hardware, including hip or knee replacement. Prior to participation in this study, informed consent was obtained for each subject in accordance with the policies and procedures of the University of Kentucky Office of Research Integrity (Lexington, KY, USA). University Institutional Review Board (IRB) approval was established to ensure that the design of this study protected the rights of the participants.

### 2.1. Anthropometric Measures

All participants were measured in lightweight clothing containing no metal and without shoes. Standing height was determined to the nearest 0.1 cm using a wall-fixed stadiometer (Healthometer Professional; Model 597KLPELSTAR; Alspin, IL, USA) with the participant’s hands positioned on the hips during a maximal inhalation. Body mass was determined to the nearest 0.01 kg using a calibrated electronic digital scale (BWB-800; Tanita Corporation, Tokyo, Japan).

### 2.2. Body Composition Measures

Body composition measures were obtained via 3 total body DXA scans and performed using a GE Lunar iDXA bone densitometer (Lunar Inc., Madison, WI, USA) during a single testing session. In accordance with state and university policies and procedures, all females of reproductive status completed a urine pregnancy test (McKesson Corp., San Francisco, CA, USA) immediately prior to DXA scanning. Only females with a negative urine pregnancy test (within the established urine-specific gravity ranges) were included.

**Figure 1:**
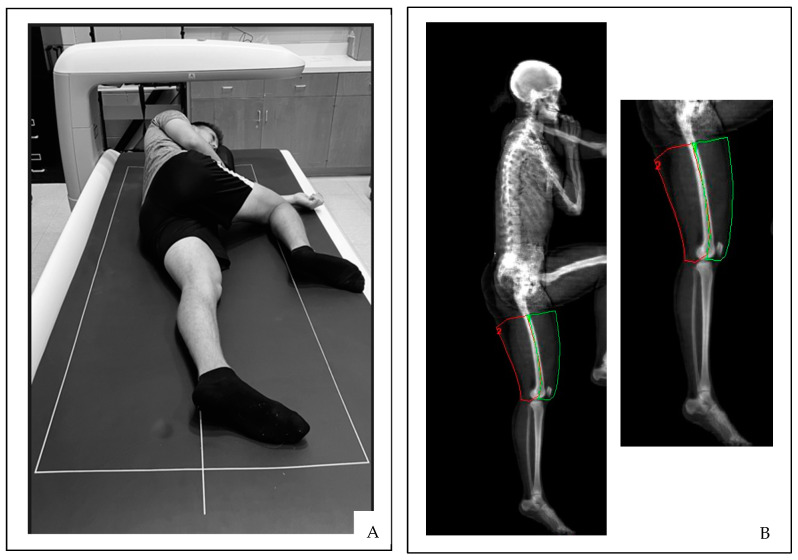
(**A**) Lateral subject positioning and (**B**) segmented body scan in the lateral view using ROI boxes.

The total body DXA scans were performed by a single trained investigator. All DXA scans were analyzed using GE Lunar software version 14.10. The first total body DXA scan was performed with the subjects lying in the standard anterior/posterior scanning position. This total body DXA scan was analyzed by the DXA scanning technologist (Investigator 1) to provide demographic information, including the total body absolute fat-free and MFL masses and the absolute and relative fat mass. Immediately following the first scan, two additional total body DXA scans were performed with the subjects lying on their left and right sides while the leg of interest was extended and the contralateral leg bent to avoid interference from the analysis field of view (Figure 1A).

Unique ROI were created for soft tissue analyses of mineral-free lean masses (MFL; g) using custom analysis software with manual tracing of the quadriceps (QUADS) and hamstrings (HAMS) of the right and left thighs. Anatomical landmarks were identified as the center of the femur (identified as the middle of the femur shaft) and soft tissue borders for the medial and lateral ROI, while the base of the gluteal fold and knee joint were used for the proximal and distal ROI boundaries (Figure 1B). These uniquely created MFL measures of the left and right QUADS and HAMS were analyzed twice by two investigators (Invest 1 and Invest 2) operating independently to provide both intra- and interrater reliability comparisons. Invest 1 was a trained and certified DXA technologist, while Invest 2 was a novice at performing DXA scan analysis.

### 2.3. Statistical Analysis

Data were analyzed using IBM SPSS Statistics (SPSS, Version 22, Armonk, NY, USA), and significance was ascribed as *p* < 0.05. Means, standard deviations (SD), and ranges for age, weight, height, body mass index (BMI), total body fat percentage (%fat), total fat mass (FM), total fat-free mass (FFM), total mineral-free lean mass (MFLM), fat-mass index (FMI) and fat-free mass index (FF MI) were determined using descriptive statistics. Descriptive statistics were also used to determine the mean, SD, and ranges for the MFLM of the right and left QUADS and HAMS, as well as the right and left total thighs for each investigator.

A series of between–within repeated measure analyses of variance (ANOVA) was used to determine if there were significant differences among the MFL measures of the right and left QUADS and HAMS and the right and left total thigh within and between Invest 1 and Invest 2. Ref. [12] plots (Bland–Altman) and correlational analyses were used to visually assess agreement and association by combining Invest 1 and Invest 2 right thigh mean versus right QUADS and HAMS combined mean and left thigh mean versus left QUADS and HAMS combined mean. Additionally, we examined the possibility of directional bias in [12] plots by quantifying the association between method differences and averages using regression. Intraclass correlation coefficients (ICC) and coefficients of variation (CV) were used to assess the intra- and interrater reliability of the segmented scans and were classified in accordance with [13].

## 3. Results

Forty-four participants (23 males) completed this study. Additional demographic, anthropometric, and body composition measures are found in Table 1. Right and left QUADS, right and left HAMS, and right and left total thigh descriptives are displayed in Table 2. Within-group analyses for Invest 1 revealed a significantly greater right QUADS mean in trial two (2338.4 ± 587.1 g) versus trial one (2316.5 ± 590.1 g), while the right HAMS mean was significantly higher in trial one (2549.1 ± 605.2 g) when compared to trial two (2518.9 ± 585.1 g) (Table 2). Invest 2 within-group analyses revealed no significant mean differences for right and left QUADS and HAMS, and right and left total thigh when comparing trial one to trial two. Between-group analyses compared mean values for Invest 1 and Invest 2. Analyses revealed that Invest 2 had significantly higher means for right QUADS (2346.6 ± 602.4 g vs. 2327.4 ± 587.9 g), left QUADS (2337.3 ± 581.9 g vs. 2312.8 ± 581.2 g), right HAMS (2655.9 ± 626.3 g vs. 2543.0 ± 593.5 g), and left HAMS (2686.1 ± 628.1 g vs. 2562.8 ± 596.5 g) when compared to Invest 1.

**Figure 2:**
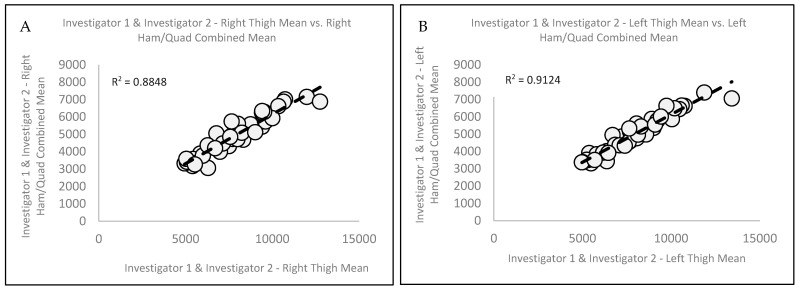
Associations between Investigator 1 and Investigator 2 combined (**A**) right thigh mean versus right HAM and QUAD combined mean and combined (**B**) left thigh mean versus left HAM and QUAD combined mean.

**Figure 3:**
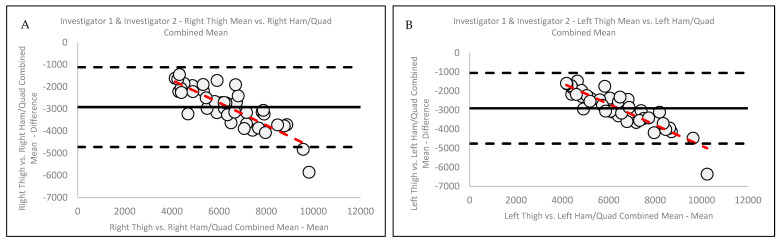
Ref. [12] plots with explained variance depicted for Investigator 1 and Investigator 2 combined (**A**) right thigh mean versus right HAM and QUAD combined mean and combined (**B**) left thigh mean versus left HAM and QUAD combined mean.

When examining the association between Invest 1 and Invest 2’s paired right thigh means and right QUADS and HAMS combined means (Figure 2A), explained variance revealed that 88.5% of the variance in the right thigh was explained by the right QUADS and HAMS combined. Similarly, when analyzing the association of Invest 1 and Invest 2′s paired left thigh means and left QUADS and HAMS combined means (Figure 2B), an analysis showed that 91.2% of the variance in the left thigh was explained by the left QUADS and HAMS combined. Ref. [12] plotting was further used to demonstrate the variability in the thigh MFL mass measurements analyses, and the resulting mean difference, ±2SD, for the right thigh versus the right QUADS and HAMS combined was −2920.9 ± 917.5 g (Figure 3A). In addition, Ref. [12] plotting indicated significant systematic (directional) bias, suggesting that as the average of the two measures increases, the difference between the two measures becomes progressively more negative for the right thigh mean. The resulting mean difference, ±2SD, for the left thigh versus the left QUADS and HAMS combined was −2905.5 ± 945.8 g (Figure 3B). Similar to Figure 3A, the [12] plot indicated significant systematic (directional) bias, suggesting that as the average of the measures increases, the difference between the two measures becomes progressively more negative for the left thigh mean.

The quantified measures of right and left QUADS and HAMS and right and left total thigh were used to assess reliability and are shown in Table 2. High interrater reliability was also demonstrated for right and left QUADS and HAMS and right and left total thigh. Interrater CV values demonstrated high reliability for right and left QUADS, right HAMS, and right and left total thigh; however, slightly lower reliability and a larger CV value were demonstrated for left HAMS. Segmented analyses resulted in strong intrarater reliability for right and left QUADS and HAMS and right and left total thighs (Table 3). Invest 1 demonstrated lower intrarater reliability than Invest 2 when comparing CV values; however, individual compartments, including right and left QUADS and HAMS, resulted in slightly lower reliability and larger CV values than right and left total thigh for both Invest 1 and Invest 2.

## 4. Discussion

There have been several previous reports indicating that both intra- and interrater reliability measures should be determined when investigating unique ROI via DXA due to subjectivity when tracing of the ROI is required and when unable to identify a specified boney landmark to standardize the boundaries of the ROI [9,10]. To date, there have been just three previously published manuscripts that have reported using DXA lateral scanning positions to assess quadriceps and hamstring soft tissue measures [5,6,9]. These three previously published manuscripts were conducted in the same laboratory and used similar DXA scanning methodologies to determine the accuracy and reliability of assessing the lateral DXA soft tissue thigh measures, to determine the association of DXA lateral leg scan MFL measures with force production, and to assess the agreement of lateral leg muscle and bone measures using DXA measures.

While our participant population (young adults), and intra- and interrater reliability results were similar to the findings of [9] there were noteworthy differences among study procedures. These differences include their use of foam pads to elevate and keep the scanned leg straight, metallic markers placed on the leg prior to scanning to assist with creating reliable analysis markers, and bone length measures to help reduce the variability in measuring the uniquely created ROI. However, the most significant procedural difference was the determination of the proximal and distal borders (determined by leg length measures) and the soft tissue ROI chosen for inclusion [9]. Furthermore, the ROI described by [9] included only a portion of the QUADS and HAMS and the lower leg MFL. Specifically, the anterior and posterior segmented upper leg compartments used 80% of the distance between the lateral epicondyle and the greater trochanter, whereas our defined regions of interest included the entire QUADS and HAMS with the distal boundary created by boney landmarks (the knee joint) and the proximal boundaries using soft tissue (the base of the gluteal fold).

We believe the greatest threat to our reliability measures was the determination of the proximal border for the ROI of the lateral positioned scans and the proximal border of the right and left HAMS MFL measures due to the lack of a boney landmark for delineation. However, incorporating the use of the iDXA software ruler function to standardize the distance from the boney landmark of the distal ROI border [9] or more precise identification of soft tissue contours may have improved the reliability of our measures. Despite having small yet significant mean differences for Invest 1, between Invest 1 and 2, and uniquely different ROI, our results revealed similar reliability measures. However, our CV% and ICC are both lower and higher, respectively, indicating greater reliability than the findings of [9] In addition, we used the same evaluative reference to classify the strength of the reliability of our ICC and CV values of our MFL measures and found that our measures were also considered highly reliable [13] Furthermore, because our method of assessing QUADS and HAMS MFL measures did not require the use of the DXA ruler software function [5,11], or placement of metallic markers on the skin of the legs to ascertain leg length measures for ROI determinations [6], we assume that our analyses are less time-consuming. We found that we could complete one trial to determine QUADS and HAMS MFL measures in less than 30 s.

The results of our [12] analysis indicated a systematic negative directional bias for both right and left thigh versus right and left QUADS and HAMS combined. Specifically, these results suggest that as the average of the measures increases, the difference between the two measures becomes progressively more negative for both the right and left thighs. While these findings suggest a consistent underestimation between analyses as the measured mass increases, the implications of these results should be considered. The findings from our [12] analyses are specific to the population studied (younger to middle-aged adults of varying adiposities); thus, these results may not be generalizable to other populations. Furthermore, these results emphasize the importance of determining standard ROI borders to increase the validity of these measures for use in clinical practice or research settings. Future studies should examine other populations to determine if alternative standardized ROIs need to be considered.

An additional factor to consider when determining the interrater reliability of DXA measures is the experience of the investigator. The impact of having multiple investigators and investigators with varying levels of experience has previously been determined for DXA body composition and bone mineral density measures [14,15]. Additionally, DXA hand-wrist scans for the assessment of skeletal maturity have also been determined [16]. The results of these studies were similar to those of the current study, which demonstrated high reliability despite the varying training status of Invest 1 and Invest 2. Thus, while minimal variability within and between investigators is desirable to improve the accuracy when these measures are compared, technician experience may not be required to produce reliable results. Based on this conclusion, we do not believe the small differences were due to the training status of the investigator but rather the lack of identifying a more standardized proximal ROI border. However, we do recommend that as new unique DXA ROI are created, the reliability of these measures needs to be determined and reported so that effect size can be more accurately determined for intervention studies.

The manufacturer, model, and software version of the DXA scanner used in this study is another factor that could provide a reason for varying results, thus necessitating the findings of this study. Prior studies conducted by [17,18] indicated that DXA image resolution and quality can vary between DXA scanner models and manufacturers. Furthermore, there are few prior studies that have noted differences in both total and regional body composition estimates between DXA models [18,19]. The model and manufacturer used in this study was a GE Lunar iDXA. While there is a large difference in the manufacturer’s proprietary analysis software used to calculate fat mass, lean mass, and bone, Ref. [20] report that the magnification effect within the GE Lunar iDXA has been corrected, thus allowing for improved image quality and resolution when creating unique ROI and when compared to other DXA models and manufacturers.

In conclusion, we believe that the DXA QUADS and HAMS measures may be a valuable tool to demonstrate associations among strength, nutritional, and training status outcomes. These measures may also provide further insight into disease progression and the effectiveness of rehabilitation strategies. We believe that we have shown that these measures can be reliably assessed without additional leg length measures or placement of external markers to determine specified boundaries; however, we still recommend that, if at all possible, a single investigator should analyze the scans twice within a given study and the mean of the two measures is used for final reporting. Additionally, it would be beneficial to encourage the DXA manufacturer software division to develop automated analysis and incorporate this into their standard analysis offerings, making it available to all iDXA users and helping further improve reliability outcomes.

## Figures and Tables

**Table 1:** Characteristics of study participants.

	Total Group (n = 44)	
Total Group (n = 44)	Mean ± SD	Range
Age (years)	23.7 ± 3.3	18–36
Weight (kg)	80.1 ± 19.4	47.5–136
Height (cm)	170.9 ± 8.8	153–187.3
Body Mass Index (kg/m^2^)	27.5 ± 6.6	19–53.4
Body Fat%	27.2 ± 11.5	9.2–62.1
Fat-Mass (kg)	22.8 ± 14.3	7.1–80.8
Fat-Free Mass (kg)	57.3 ± 12.6	38.1–86.3
Mineral-Free Lean Mass (kg)	54.2 ± 12.1	35.8–86.3
Fat-Mass Index (kg/m^2^)	7.9 ± 5.6	2.1–33.2
Fat-Free Mass Index (kg/m^2^)	19.4 ± 2.9	14.5–27.5

**Table 2:** Quadriceps, hamstrings, total thigh descriptives and ANOVA results.

			Investigator 1						Investigator 2			
	Trial One		Trial Two		Mean		Trial One		Trial Two		Mean	
Compartment	Mean ± SD	Range	Mean ± SD	Range	Mean ± SD	Range	Mean ± SD	Range	Mean ± SD	Range	Mean ± SD	Range
Right Quadricep (g)	2316.5 ± 590.1	1401–3505	2338.4 ± 587.1 *	1423–3502	2327.4 ± 587.9	1418.5–3503.5	2349.2 ± 606.5	1485–3520	2344.1 ± 598.7	1486–3493	2346.6 ± 602.4 ^a^	1485.5–3506.5
Left Quadricep (g)	2303.9 ± 578.4	1380–4063	2321.7 ± 587.7	1356–3955	2312.8 ± 581.2	1368–4009	2338.9 ± 583.8	1372–4097	2335.8 ± 580.5	1398–4046	2337.3 ± 581.9 ^a^	1385–4071.5
Right Hamstring (g)	2549.1 ± 605.2 *	1527–3808	2518.9 ± 585.1	1523–3646	2543.0 ± 593.5	1554–3727	2657.0 ± 620.7	1617–3848	2654.8 ± 632.4	1609–3935	2655.9 ± 626.3 ^a^	1620–3882
Left Hamstring (g)	2568.5 ± 539.9	1612–3698	2557 ± 603.5	1656–3933	2562.8 ± 596.5	1644.5–3815.5	2689.5 ± 630.8	1644–4042	2682.8 ± 625.9	1660–4032	2686.1 ± 628.1 ^a^	1652–4037
Right Total Thigh (g)	7856.7 ± 1929.1	4893–12,928	7831.6 ± 1869.9	4914–12,608	7844.2 ± 1895.9	4903.5–12,768	7868.7 ± 1955.9	4982–12,767	7854.8 ± 1930.9	4985–12,662	7861.8 ± 1943.2	4983.5–12,714.5
Left Total Thigh (g)	7884.8 ± 2027.6	4844–13,694	7884.8 ± 1966.7	4963–13,616	7884.8 ± 1994.7	4948–13,655	7828.7 ± 1954.7	4959–13,162	7822.2 ± 1956.0	5021–13,207	7825.4 ± 1955.1	4990–13,184.5

* *p* < 0.05: Within Group; Trial one versus Trial two; ^a^ *p* < 0.05; Between Group; Investigator 1 Mean versus Investigator 2 Mean. Abbreviations: SD = standard deviation.

**Table 3:** Interrater and intrarater reliability CV for quadriceps, hamstrings, and total thigh.

	Interrater Coefficients		Intrarater Coefficients			
			Investigator 1		Investigator 2	
Compartment	CV	ICC	CV	ICC	CV	ICC
Right Quadricep (g)	0.88	0.998	1.13	0.997	0.52	0.999
Left Quadricep (g)	0.13	0.997	1.61	0.993	0.52	0.999
Right Hamstring (g)	0.14	0.985	1.56	0.994	0.59	0.999
Left Hamstring (g)	2.41	0.984	1.6	0.992	0.51	0.999
Right Total Thigh (g)	0.96	0.997	0.92	0.996	0.30	1.000
Left Total Thigh (g)	0.07	0.997	0.99	0.997	0.30	1.000

‘Right total thigh’ and ‘left total thigh’ refer to the sum of the quadricep and hamstring compartment for each measure of each leg (right and left). Abbreviations: CV = coefficients of variation; ICC = intraclass correlation coefficients.

## Data Availability

The datasets generated during and/or analyzed during the current study are not publicly available due to the University of Kentucky’s data retention and ownership policy.
